# Assessment of Patient Journey Metrics for Users of a Digital Obstructive Sleep Apnea Program: Single-Arm Feasibility Pilot Study

**DOI:** 10.2196/31698

**Published:** 2022-01-12

**Authors:** Shefali Kumar, Emma Rudie, Cynthia Dorsey, Amy Blase, Adam V Benjafield, Shannon S Sullivan

**Affiliations:** 1 Verily Life Sciences South San Francisco, CA United States; 2 ResMed Corporation San Diego, CA United States

**Keywords:** obstructive sleep apnea, virtual care, remote care, OSA diagnosis, sleep apnea, OSA, underdiagnosed, feasibility, patient-centered, treatment pathway, diagnostic, eHealth

## Abstract

**Background:**

Despite the importance of diagnosis and treatment, obstructive sleep apnea (OSA) remains a vastly underdiagnosed condition; this is partially due to current OSA identification methods and a complex and fragmented diagnostic pathway.

**Objective:**

This prospective, single-arm, multistate feasibility pilot study aimed to understand the journey in a nonreferred sample of participants through the fully remote OSA screening and diagnostic and treatment pathway, using the Primasun Sleep Apnea Program (formally, Verily Sleep Apnea Program).

**Methods:**

Participants were recruited online from North Carolina and Texas to participate in the study entirely virtually. Eligible participants were invited to schedule a video telemedicine appointment with a board-certified sleep physician who could order a home sleep apnea test (HSAT) to be delivered to the participant's home. The results were interpreted by the sleep physician and communicated to the participant during a second video telemedicine appointment. The participants who were diagnosed with OSA during the study and prescribed a positive airway pressure (PAP) device were instructed to download an app that provides educational and support-related content and access to personalized coaching support during the study’s 90-day PAP usage period. Surveys were deployed throughout the study to assess baseline characteristics, prior knowledge of sleep apnea, and satisfaction with the program.

**Results:**

For the 157 individuals who were ordered an HSAT, it took a mean of 7.4 (SD 2.6) days and median 7.1 days (IQR 2.0) to receive their HSAT after they completed their first televisit appointment. For the 114 individuals who were diagnosed with OSA, it took a mean of 13.9 (SD 9.6) days and median 11.7 days (IQR 10.1) from receiving their HSAT to being diagnosed with OSA during their follow-up televisit appointment. Overall, the mean and median time from the first televisit appointment to receiving an OSA diagnosis was 21.4 (SD 9.6) days and 18.9 days (IQR 9.2), respectively. For those who were prescribed PAP therapy, it took a mean of 8.1 (SD 9.3) days and median 6.0 days (IQR 4.0) from OSA diagnosis to PAP therapy initiation.

**Conclusions:**

These results demonstrate the possibility of a highly efficient, patient-centered pathway for OSA workup and treatment. Such findings support pathways that could increase access to care, reduce loss to follow-up, and reduce health burden and overall cost. The program’s ability to efficiently diagnose patients who otherwise may have not been diagnosed with OSA is important, especially during a pandemic, as the United States shifted to remote care models and may sustain this direction. The potential economic and clinical impact of the program’s short and efficient journey time and low attrition rate should be further examined in future analyses. Future research also should examine how a fast and positive diagnosis experience impacts success rates for PAP therapy initiation and adherence.

**Trial Registration:**

ClinicalTrials.gov NCT04599803; https://clinicaltrials.gov/ct2/show/NCT04599803

## Introduction

Obstructive sleep apnea (OSA) is a prevalent sleep-related breathing disorder affecting up to 1 billion people worldwide [1]. OSA is associated with many common and costly comorbidities such as hypertension, diabetes, stroke, asthma, depression, and other cardiovascular and cerebrovascular conditions [2]. Untreated OSA is associated with an increased risk of morbidity and mortality and can result in the worsening of comorbid conditions as well as diminished quality of life [2,3]. Untreated OSA may also be associated with motor vehicle accidents and accidents in the workplace [4]. In addition to the impact on clinical outcomes, untreated OSA is associated with increased health care utilization and can result in increased direct and indirect costs (eg, workplace productivity, absenteeism, and presenteeism) [5,6].

Given the potential clinical and economic impact, it is important for individuals with OSA to be diagnosed efficiently, to receive treatment, and to stay adherent to treatment [7,8]. For most patients with moderate-to-severe OSA, first-line therapy is positive airway pressure (PAP) therapy [9]. Despite the importance of diagnosis and treatment, OSA remains a vastly underdiagnosed condition; this is partially due to current OSA identification methods and a complex and fragmented diagnostic pathway [10]. Traditionally, polysomnography (PSG) has been used to diagnose OSA, which requires a patient to stay overnight at a sleep clinic [6]. The traditional pathway is labor-intensive, requires a great deal of effort and proactiveness by the patient, and is dependent on sleep lab capacity and staffing. Home sleep apnea tests (HSATs) are a viable alternative to PSG, as they reliably and cost-efficiently diagnose OSA, do not require patients to stay overnight at a sleep clinic, and are less expensive than PSG [7,11]. Even so, a variety of considerations such as appointment availability, challenges surrounding in-person appointments for evaluation, picking up, and dropping off testing equipment, and prolonged waits to receive results may impact progress through diagnostic and treatment pathways [12,13]. Furthermore, education about OSA and support for patients during the diagnostic process are often lacking. Taken together, the numerous steps and the involvement of multiple health care providers in the traditional pathway predispose to a fractured experience, high attrition rates, and barriers to OSA diagnosis and treatment in a timely and patient-centered manner.

With the advancement of telemedicine and digital technologies, virtual care methods are increasingly being used to diagnose and treat sleep disorders [14,15]. The COVID-19 pandemic rapidly catalyzed the adoption of telemedicine and nontraditional health care delivery methods across the United States [16,17]. Although there is an increase in the use of telemedicine and virtual care methods for OSA diagnosis and disease management, additional research is required to understand whether clinical outcomes for patients who receive care virtually differ from the outcomes for patients who receive it through traditional means (eg, in-person visits) [15]. One recent review and meta-analysis found that, based on the results from 16 randomized controlled trials, telemedicine interventions were associated with increased PAP usage and PAP adherence for individuals with OSA [18].

A distinctive feature of the Primasun Sleep Apnea Program (PSAP) is that it can be applied to general clinical populations, which guides individuals completely virtually, with no in-person visits, through the OSA diagnosis process, onboarding them onto therapy and providing them with continuous support and care once therapy has been initiated. The patient journey experienced by the users of this program parallels what is experienced in traditional clinical practice, but by using a health information technology solution (including virtual appointments, an app, and a website). This platform focuses on improving the patient experience, promoting clinical efficacy, and reducing attrition rates through the diagnostic pathway. Independent features of this platform have also been proposed to increase access to care. This study was undertaken to understand the journey in a nonreferred sample of participants through a fully remote OSA screening and diagnostic and treatment pathway.

The objectives of this single-arm feasibility pilot study were to assess patient journey metrics, including time to physician evaluation and testing, as well as time to initiate therapy if recommended by the sleep physician, patient satisfaction, and program completion rates for users of this entirely virtual program.

## Methods

### Study Design

This prospective, single-arm, multistate feasibility pilot study of a fully virtual OSA assessment, diagnostic, and management platform aimed to assess the patient journey (eg, platform ease of use, time to diagnosis, time to initiate PAP therapy if clinically recommended, and pathway completion rates) as well as satisfaction. Study duration differed for each participant; duration depended on how long it took the participant to complete the diagnostic pathway and whether the participant continued onto the PAP therapy portion of the study. The PAP therapy study period was 90 days. The study followed the STROBE (Strengthening the Reporting of Observational Studies in Epidemiology) guidelines [19].

This study was conducted entirely virtually using the Baseline Platform, a comprehensive remote clinical studies platform for recruitment, consenting, screening, enrollment, data collection, and study monitoring. This study was approved by the Western Institutional Review Board and is registered on ClinicalTrials.gov (NCT04599803).

### Program Overview

The virtual platform investigated in this study was the PSAP platform, which is intended to support and educate individuals at risk for OSA as they navigate the complex, multistep assessment and diagnosis pathway in a more patient-centered, efficient, and entirely virtual way. The program facilitates preassessment, online scheduling, one-on-one video televisits with board-certified sleep physicians pretesting and posttesting, and an entirely remote diagnostic testing process for OSA. Education on healthy sleep and OSA is provided, and patients can reach out to a dedicated support team with questions at any point during the assessment and diagnostic process.

For cases in which OSA is diagnosed and PAP is prescribed as therapy, the PSAP is also intended to assist in getting the patient equipped with a PAP device and supplies, provide a supportive onboarding experience onto PAP therapy, address questions and concerns with real-time support, and help patients to become adherent to therapy. Health coaches are available to engage in two-way communications through the platform to provide educational information related to sleep, OSA, and PAP therapy, as well as to use motivational enhancement techniques and general support.

### Recruitment and Study Eligibility

Study participants were recruited through online registries and targeted digital advertisements in North Carolina and Texas. North Carolina and Texas were selected as study locations because of practical considerations around physician availability and related elements. Potential participants were first provided with a study overview on a web landing page. On this page, they were also given the option to consent to the Project Baseline Community Study [20]. Project Baseline Community Study is an online registry for individuals who are interested in opportunities to participate in health-related research, test new technology, and learn about their health. Once enrolled in the Project Baseline Community Study, the participant could proceed to an online preassessment tool to assess initial eligibility. This tool included the OSA-50, an established 4-question validated OSA screening questionnaire [21], as well as questions assessing shift worker status, pregnancy, supplemental oxygen use, and verification of access to platform-compatible devices. Individuals who appeared to be qualified were then asked to review and sign the study’s specific informed consent form to participate in the study.

To be eligible for the study, the participants were required to be 18 years or older, speak and read English, live in North Carolina or Texas, own a compatible smartphone, have access to a computer with a camera, and have consistent access to electricity and internet. The participants were also required to have a high risk of OSA, based on the OSA-50 questionnaire (score of 5 or greater) [21]. The participants were excluded if they reported being previously diagnosed with OSA or certain other chronic sleep disorders (eg, central sleep apnea, complex sleep apnea, or chronic insomnia); being a shift worker; being or planning to become pregnant during the study period; being employed by the sponsor or by individuals working on Baseline Community; or using a home supplemental oxygen device.

To be eligible to continue onto the PAP therapy portion of the study, the participants must have completed the post-HSAT sleep medicine physician televisit by December 31, 2020. Additionally, they needed to have been diagnosed with OSA and prescribed PAP therapy by their sleep study physician.

### Procedures

Once deemed eligible, the participant completed a medical and sleep history questionnaire covering medical and sleep information as well as an Epworth Sleepiness Scale (ESS) for the sleep physician to review [22]. After completing the questionnaires, the participant was invited to schedule their first sleep televisit appointment using an online scheduling tool. During the first televisit appointment, the physician performed a sleep medicine consultation. If the sleep physician ordered HSAT as a result of the consultation, the HSAT order was placed through the PSAP platform.

The HSAT device used for this study was WatchPat One (Itamar Medical Ltd). Following HSAT completion, the patients received an emailed invitation to schedule a follow-up physician video telemedicine appointment to review HSAT results and plan for further care as appropriate and recommended by the sleep physician. At appropriate time points, the participants who were not suspected to have OSA, were suspected to have conditions requiring in-laboratory PSG testing, were not diagnosed with OSA, were recommended non-PAP treatment options, or chose not to proceed with PAP therapy were exited from the study and connected to additional local clinical resources when needed and appropriate.

The participants who were diagnosed with OSA during the study and prescribed a PAP device were instructed to download the PSAP app, which provides educational and support-related content. The PSAP app also guides the participants through the device-ordering and mask-selection process and schedules the delivery of a welcome kit, which includes comprehensive tools for starting PAP therapy, guiding and supporting the participants to successfully adhere to therapy.

The participant could access the tools and resources in the PSAP app to attain set-up support and begin using the PAP device. As the participants used the device over the next 90 days, they could reach out to health coaches via messaging with any questions or support they needed. The participants also had access to phone support during business hours.

There were 5 study surveys administered during the study period. These surveys assessed baseline demographic information, knowledge of sleep apnea prior to diagnosis, and satisfaction with the program. After the 90-day PAP therapy study period, the participants conducted a final study exit televisit appointment where they were provided guidance on how to continue therapy (if desired) outside of the study.

### Outcomes

The primary objective of this study was to assess patient journey metrics using a fully remote system for the diagnosis of OSA and onboarding of PAP therapy. The analysis presented in this publication focuses on the cohort of individuals who initiated the diagnostic pathway and examines the following key patient journey outcomes: time from the first televisit appointment to OSA diagnosis; time from OSA diagnosis to PAP therapy initiation; time from the first televisit appointment to when the patient receives the HSAT; time from when the participant receives the HSAT to OSA diagnosis; and the percent of individuals who completed various stages of the diagnosis pathway.

Usability of the platform, as measured by retention and survey feedback, and program satisfaction were among some of the exploratory objectives of the study, which will be presented in this manuscript. Participant satisfaction with the program overall and with certain program features was measured using a standard 5-point Likert scale ranging from “very dissatisfied” to “very satisfied.”

### Sample Size and Statistical Analyses

Given the preliminary nature of this pilot, we did not power the study to measure a specific difference in any of the outcomes. Our goal was to have a minimum of 50 individuals on PAP therapy.

Mean and standard deviation or proportions, when relevant, are reported for baseline demographics and clinical characteristics. For patient journey time metrics, the mean, standard deviation, median, and interquartile range are reported, and outliers examined. Moreover, attrition rates (ie, the proportion of individuals who dropped out of the diagnostic pathway) for various points in the diagnostic pathway are reported. All analyses were conducted in Python (version 3.6.13; Python Software Foundation).

## Results

### Study Sample

**Figure 1:**
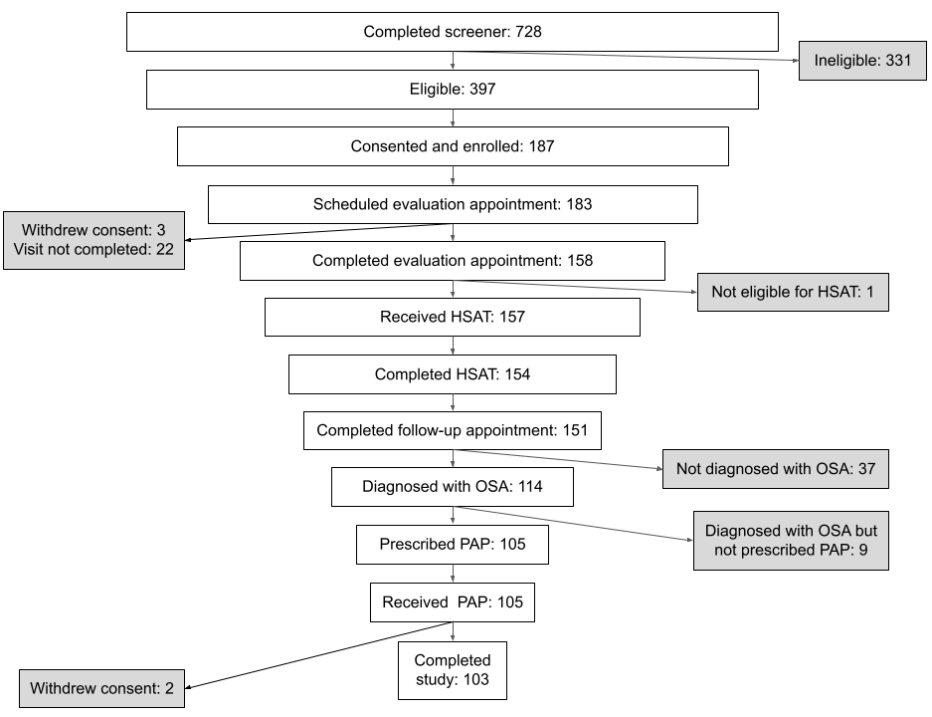
Participant flow through the pilot study; HSAT: home sleep apnea test; OSA: obstructive sleep apnea; PAP: positive airway pressure.

Figure 1 details recruitment and participant flows through the study. Study recruitment, screening, and enrollment took place between June 2020 and October 2020. Of the 687 individuals who completed the online screening questionnaire for eligibility, 57.8% (n=397) were eligible. A total of 71.5% (n=284) of the eligible individuals provided electronic informed consent for the study. Of the 284 individuals who provided consent, 85.9% (n=255) enrolled in the study. A total of 71.8% (n=183) of the enrolled participants scheduled their first televisit appointment. Of the 183 individuals who scheduled a first televisit appointment, 86.3% (n=158) completed their appointment; there were 25 individuals who initially scheduled an appointment but either canceled their appointment and never rescheduled or missed their first scheduled televisit appointment and never successfully completed it. Of the 158 individuals who completed their first televisit appointment, 99.4% (n=157) were determined appropriate for HSAT by the sleep medicine physician. Moreover, 1 individual was referred out of the study to in-laboratory testing for clinical suspicion of a different disorder. Of the 157 individuals who received an HSAT, 98.1% (n=154) successfully completed the HSAT and 96.2% (n=151) completed their follow-up televisit appointment. A total of 114 individuals were diagnosed with OSA, and 105 were prescribed PAP therapy. Of the 105 individuals who were prescribed PAP therapy as part of this program, all were able to download the PSAP app to order the PAP device. All 105 individuals received a PAP device during the study.

### Baseline Demographic and Clinical Characteristics

An overview of baseline demographics and self-reported clinical characteristics for the 158 individuals who completed the first televisit appointment is reported in Table 1. The majority of this population were female (95/158, 60%), White (141/158, 89%), had an associate degree or higher (82/142, 58%), and were from Texas (103/158, 65%). The mean age of the population was 47 years (SD 10, range 24-72 years). At baseline, the participants reported an average ESS of 10.7 (SD 4.8), with about half of the study population having an ESS greater than 10 (80/158, 51%). Average BMI was 34.6 kg/m^2^ (SD 8.3), and the majority of the population (105/158, 66%) were obese (BMI of at least 30 kg/m^2^). Top comorbidities included allergies (76/158, 46%), high blood pressure (60/158, 38%), and diabetes (30/158, 19%). A majority of the population had health insurance (107/142, 75%), had a primary care physician (107/158, 68%), and were at least somewhat familiar with sleep apnea prior to enrolling in the study (130/142, 92%). Approximately 6% (9/158) of this population had completed a sleep study previously.

**Table 1:** Key baseline demographics and self-reported clinical characteristics of those who initiated the diagnostic pathway (n=158).

Characteristics		Values
Age (years), mean (SD)		46.9 (10.4)
**Sex, n (%)**		
	Female	95 (60)
	Male	63 (40)
**Race or ethnicity, n (%)^a^**		
	White	141 (89)
	Black	8 (5)
	Hispanic	30 (19)
	Asian	7 (4)
	Native American	4 (3)
	Other	6 (4)
**Geographic location, n (%)**		
	North Carolina	55 (35)
	Texas	103 (65)
**Comorbidities, n (%)**		
	Allergies	76 (46)
	High blood pressure	60 (38)
	Diabetes	30 (19)
	Bruxism	26 (17)
	Swollen legs	20 (13)
	Bronchitis or asthma	19 (12)
	Thyroid issues	16 (10)
	Migraine	16 (10)
	Anemia	15 (9)
	Dizziness or fainting	12 (8)
	Chronic pain	11 (7)
	Heart murmur	9 (6)
	Temporomandibular joint syndrome	9 (6)
	Arrhythmia	8 (5)
	Mononucleosis	8 (5)
	Concussion	7 (4)
	Coronary artery disease	6 (4)
	Epilepsy or seizures	4 (3)
	Stroke	3 (2)
	Heart attack	3 (2)
	COPD^b^	3 (2)
	Cognitive impairment	1 (1)
	Other lung disease	1 (1)
	Heart failure	1 (1)
BMI (kg/m^2^), mean (SD)		34.6 (8.3)
**BMI (kg/m^2^), n (%)**		
	<18.5	0 (0)
	18.5 to <25	13 (8)
	25 to <30	40 (25)
	30 to <35	35 (22)
	35+	70 (44)
OSA-50^c^, mean (SD)		7.2 (1.6)
ESS^d^, mean (SD)		10.7 (4.8)
**ESS, n (%)**		
	0-5	30 (19)
	6-10	48 (30)
	11-12	19 (12)
	13-15	41 (26)
	16-24	20 (13)

^a^Does not sum to 100% because the participants were able to select multiple answer choices.

^b^COPD: chronic inflammatory lung disease.

^c^A validated obstructive sleep apnea questionnaire.

^d^ESS: Epworth Sleepiness Scale.

### Patient Journey Metrics

**Figure 2:**
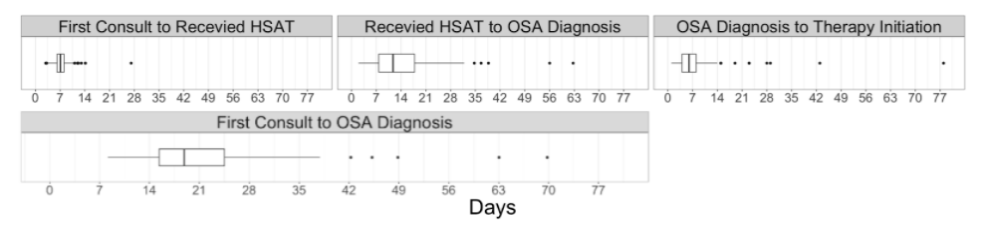
Outlier detection for key patient journey time metrics. HSAT: home sleep apnea test; OSA: obstructive sleep apnea.

**Figure 3:**
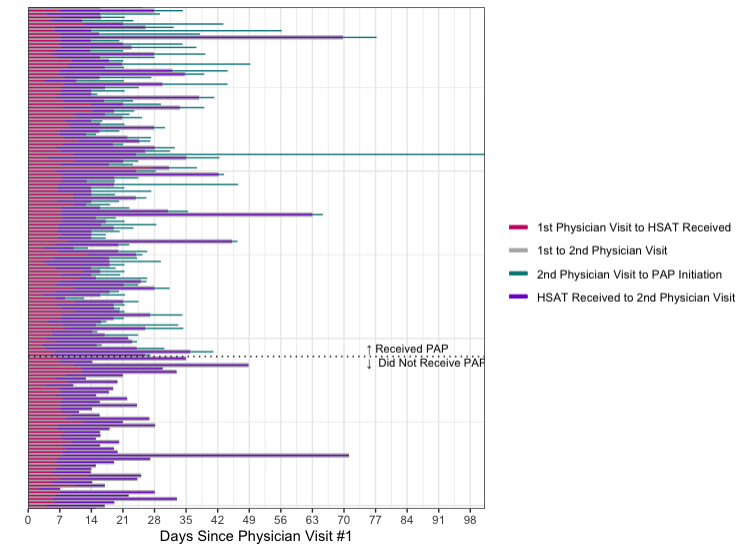
Key patient journey time metrics. HSAT: home sleep apnea test; PAP: positive airway pressure.

**Figure 4:**
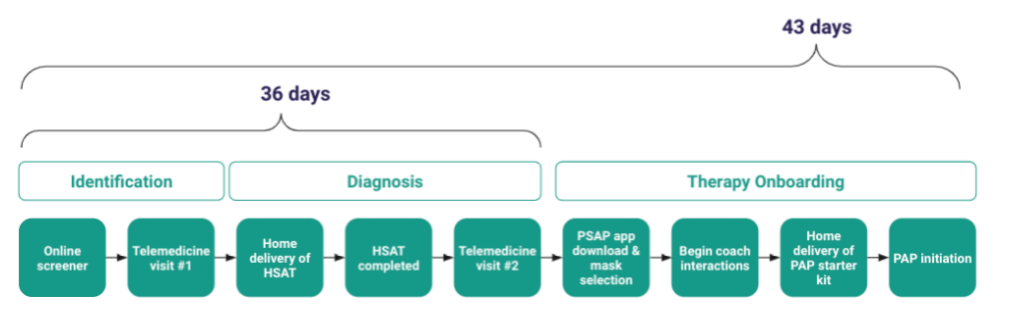
Median total journey times in obstructive sleep apnea (from screening to onboarding to positive airway pressure). HSAT: home sleep apnea test; PSAP: Primasun Sleep Apnea Program; PAP: positive airway pressure.

The main journey time results for this pilot are depicted in Table 2. For the 157 individuals who were ordered an HSAT, it took a mean of 7.4 (SD 2.6) days and median 7.1 days (IQR 2.0) to receive their HSAT after they completed their first sleep medicine televisit appointment with the physician. For the 114 individuals who were diagnosed with OSA, it took a mean of 13.9 (SD 9.6) days and median 11.7 days (IQR 10.1) from receiving their HSAT to receiving a diagnosis of OSA from their physician at their follow-up televisit appointment. Of those who were diagnosed with OSA (n=114), mean journey times were 21.4 (SD 9.6) days from the first physician televisit appointment to the 2nd physician televisit when they received the diagnosis; and 8.1 (SD 9.3) days from the diagnosis until receiving their PAP device, if prescribed. Median journey times were explored as well in order to best assess for outliers (defined as 1.5 times IQR above the 75th percentile) (Figure 2). All 4 metrics show skewness of the distributions due to outliers, which were preserved in this analysis to reflect real-world scenarios such as shipping delays, holidays, and multiple missed appointments. As such, these statistics reflect realistic distributions of patient journey times. There were no patterns detected in journey times over the course of the study. There was no significant difference in journey times between those with and without OSA diagnosis (*P*=.93 using the Welch *t* test) (Figure 3). Overall, median patient journey times from first landing on the screening webpage to onboarding to PAP therapy were approximately under 6 weeks (Figure 4).

Figure 2 demonstrates median, interquartile range, and outlier data (in days) for key journey metrics.

The chart in Figure 3 depicts the key journey time metrics for each participant, with the length of the bar representing the number of days elapsed since 1st physician visit. The bars are ordered from top to bottom in chronological order for the first physician appointment.

Figure 4 displays the median total journey times (in days) for the participants, from first landing on the screening website to completing 2 sleep physician televisits plus sleep testing, and to starting PAP therapy if diagnosed with OSA and recommended for PAP therapy.

**Table 2:** Mean, median, and range for key participant journey metrics (in days).

Milestone	Mean (SD)	Median (IQR)	Range
Days from the 1st physician visit to the 2nd physician visit (post-HSAT^a^) (n=151)	21.1 (9.8)	18.8 (9.0)	7.1-71.1
Days from the 1st physician visit to receiving HSAT (n=157)	7.4 (2.6)	7.1 (2.0)	2.1-27.1
Days from OSA^b^ diagnosis (2nd physician visit) to PAP^c^ therapy initiation (n=105)	8.1 (9.3)	6.0 (4.0)	1-78

^a^HSAT: home sleep apnea test.

^b^OSA: obstructive sleep apnea.

^c^PAP: positive airway pressure.

### Program Satisfaction

A total of 100 participants (all with OSA, 99 of whom went on to use the PSAP app) provided satisfaction scores after the 2nd physician televisit. The mean satisfaction rating for the diagnostic portion of the program was 4.75 (SD 0.67) out of 5, corresponding to a “very satisfied” rating. Overall, 84% (n=84) of the participants provided a 5-out-of-5 satisfaction rating, with 2% (n=2) rating less than a 3 out of 5. In addition, 86% (n=86) of the participants reported that registering for the program was very easy (average rating of 4.78 out of 5, SD 0.65), and 77% (n=77) were very satisfied with the onboarding process (average rating of 4.69 out of 5, SD 0.69).

A total of 76 participants provided total program satisfaction scores at the conclusion of 90 days of PAP on our study. The mean satisfaction score for these participants was 4.3 (SD 1.0), and the median score was 5, corresponding to a “very satisfied” rating, with 6.6% of the participants (n=5) providing a rating of less than 3 out of 5. Overall, 81.6% (62/76) of the participants provided a 4 or 5 out of 5, corresponding to a “satisfied” or “very satisfied” rating.

## Discussion

### Principal Findings

Overall, the participants in our program were able to quickly and efficiently complete the OSA diagnostic pathway using PSAP. It took approximately 3 weeks from the initial televisit to OSA diagnosis and an additional 1 week from diagnosis to PAP therapy initiation. This includes scheduling and completing 2 televisit appointments, receiving, completing, and sending back an HSAT, the interpretation of the HSAT results by a board-certified sleep physician, and ordering and receiving a PAP device and the associated supplies. For purposes of comparison, the length of time it takes an individual to complete the OSA diagnostic pathway under standard of care is highly variable in the United States and largely depends on geographic location, physician availability, type of sleep test performed (HSAT or PSG), referral method, and other factors. Estimates show that it can take anywhere from weeks to over a year for an individual to eventually get diagnosed with OSA and initiate therapy in the United States [23]. For example, according to one analysis of a sleep center in Philadelphia, OSA diagnosis may take from 4 weeks to 8 months from initial referral to sleep evaluation and then an additional time for PAP initiation. The Veterans Health Administration facilities report that it can take about 8 to 9 months on average from initial referral to sleep evaluation [23]. One study examining a home management pathway for OSA in Canada found that the home management program reduced wait times from 152 days to 92 days, on average [24]. In the United Kingdom, the average wait time from initial physician referral to PAP initiation is approximately 14 months [23]. Therefore, compared to the limited evidence available, this program’s patient journey times are promising.

Attrition in traditional models of sleep care at each step of the pathway is also variable and underreported. In our study, among those who screened into an offer for an initial sleep physician evaluation and made an appointment through the platform, 86.3% completed the appointment. This is a no-show or cancellation rate that compares favorably to reports describing this journey in clinical practice. For example, a recently published retrospective chart review of over 2000 sleep clinic visits found a no-show rate of 30.5% for the first clinic visit and an overall no-show rate of 21% [25]. The better attendance rate seen in this study may be related in part to the older mean age of the group and the fact that most had insurance, which may reflect being more connected to health care opportunities, generally. Moreover, long waits for appointments may traditionally drive no-shows, and delays in testing and diagnosis complicate the patient journey and interfere with ultimate treatment success [26]. In PSAP, appointments were available typically within 1-2 weeks at a variety of times including outside traditional office hours, and email reminders were sent prior to the appointments. The shorter wait times and email reminders may have been factors in the high show rates observed in this study.

Similarly, completion rates for HSAT were extremely high. Moreover, the completion rate for the 2nd physician televisit, the HSAT “results” televisit, was very high, at 98%. Though data are sparse on this metric in the published literature, the authors believe that the less-than-2% attrition following HSAT may indicate an ease-of-use advantage for clinical care.

Overall, these results are very promising, especially given that this study was conducted during the COVID-19 pandemic, an era marked by uncertainty, competing priorities, limited access to health care, and illness, not to mention delays in shipping and reduced supply availability. The program was able to effectively reach individuals and had very low attrition rates, with less than 5% of the study population lost to follow-up during the diagnostic period after completing their first appointment. The program received very positive feedback; most of the participants were satisfied to very satisfied with their experience with the program and the program’s ability to guide them through the complex OSA diagnostic pathway. This is especially meaningful considering world events during this study, which included the COVID-19 pandemic and related occupational economic, social upheavals which have been reported to interrupt medical care, as well as the widespread power outages in Texas due to weather in February 2021 [27].

### Strengths and Limitations

One of the major strengths of this study was its fully virtual nature. This allowed the study to mimic real-world settings as the study participants did not need to visit in-office clinics to complete study documents and provide study data. Instead, they interacted with PSAP as they would have outside the study. Our analyses also include all outliers from the study to appropriately demonstrate how the program may perform in the real world.

There were some limitations to this study. First, given that this was designed as a single-arm study with no control population, a direct comparison of the study participants’ patient journey experience through the OSA diagnostic pathway with standard of care was not possible. But as previously mentioned, standard of care is extremely variable, so a true generalizable control would be difficult to construct and examine. Despite not having a control group, the authors believe these results are extremely useful when assessing the value of this program. The study population was also primarily female and White, and all participants were from North Carolina or Texas. Most participants reported having health insurance and a primary care doctor. Therefore, the results may not be fully generalizable to other populations. Future studies should examine the program in a more diverse population. The participants were also identified and recruited for the study through digital means, which may mean that our study population is not fully generalizable to the entire undiagnosed OSA population in the United States. However, given that the PSAP is a digital and app-based solution, this population may represent a new subgroup of the undiagnosed OSA population who would benefit from this solution and who may not necessarily be reached by standard care pathways. Regarding satisfaction scores, only the participants who were diagnosed with OSA or those who completed the app portion of the journey (for PAP treatment) filled out satisfaction surveys. Therefore, it is not possible to ascertain the degree of satisfaction with the program in the subgroup of participants who were not diagnosed with OSA in this pathway, or who were diagnosed with OSA and were recommended treatments other than PAP therapy. This area certainly requires further study.

### Conclusions

In this single-arm feasibility pilot study, we assessed the patient journey for users of a fully virtual diagnosis and OSA management program. The results of this pilot study are promising when considering increasing access to care, improving inefficiencies and inconsistencies in diagnostic pathways, and reducing overall costs. The program’s ability to diagnose patients who otherwise may have not been diagnosed with OSA is extremely important, especially during a pandemic as the country shifted to virtual and remote care models and may sustain this direction. The potential economic and clinical impact of the program’s short and efficient journey time and low attrition rate should be further examined in future analyses. Future research also should examine how an efficient and positive diagnosis experience impacts success rates for PAP therapy initiation and long-term adherence.

## Abbreviations

ESS: Epworth Sleepiness Scale
HSAT: home sleep apnea test
OSA: obstructive sleep apnea
PAP: positive airway pressure
PSAP: Primasun Sleep Apnea Program
PSG: polysomnography
STROBE: Strengthening the Reporting of Observational Studies in Epidemiology
